# Mitochondrial-Derived Vesicles—Link to Extracellular Vesicles and Implications in Cardiovascular Disease

**DOI:** 10.3390/ijms24032637

**Published:** 2023-01-30

**Authors:** Jonas Heyn, Marina Augusto Heuschkel, Claudia Goettsch

**Affiliations:** Department of Internal Medicine I—Cardiology, Medical Faculty, RWTH Aachen University, 52074 Aachen, Germany

**Keywords:** mitochondria, mitochondrial-derived vesicles, extracellular vesicles, mitochondrial transfer, cardiovascular disease

## Abstract

Mitochondria are dynamic organelles regulating metabolism, cell death, and energy production. Therefore, maintaining mitochondrial health is critical for cellular homeostasis. Mitophagy and mitochondrial reorganization via fission and fusion are established mechanisms for ensuring mitochondrial quality. In recent years, mitochondrial-derived vesicles (MDVs) have emerged as a novel cellular response. MDVs are shed from the mitochondrial surface and can be directed to lysosomes or peroxisomes for intracellular degradation. MDVs may contribute to cardiovascular disease (CVD) which is characterized by mitochondrial dysfunction. In addition, evidence suggests that mitochondrial content is present in extracellular vesicles (EVs). Herein, we provide an overview of the current knowledge on MDV formation and trafficking. Moreover, we review recent findings linking MDV and EV biogenesis and discuss their role in CVD. Finally, we discuss the role of vesicle-mediated mitochondrial transfer and its potential cardioprotective effects.

## 1. Introduction

As the organelles responsible for producing adenosine triphosphate (ATP), mitochondria are the cellular powerhouse. A healthy heart consumes approximately 6 kg of ATP per day, 95% of which is produced by mitochondria [1,2]. To successfully meet this enormous energy demand, mitochondria occupy roughly one-third of the volume of an adult cardiomyocyte [3]. In addition to cellular energy metabolism, mitochondria control calcium homeostasis, viability, and redox status [2].

The number of mitochondria and their activity can change in response to various physiological or pathological conditions, demonstrating that they are highly dynamic organelles. The balance of mitochondrial fusion, fission, and mitophagy characterizes this constant mitochondrial remodeling. In contrast to mitochondrial fission, which divides a single mitochondrion into two or more distinct organelles, mitochondrial fusion is the joining of the outer and inner mitochondrial membranes of two different mitochondria. Mitophagy requires mitochondrial fission to separate damaged mitochondrial segments that are sequestered, delivered to, and degraded in the lysosomes [4,5].

Mitochondrial dysfunction and dysregulation of mitochondrial remodeling processes play critical roles in the pathogenesis of cardiovascular disease (CVD) [2]. Oxidative stress, a byproduct of mitochondrial dysfunction, has been identified as a risk factor for vascular dysfunction and CVD [6]. Therefore, mitochondria-targeted therapeutic strategies have received increased attention. However, generic antioxidants failed to improve CVD outcomes in clinical trials, possibly due to delivery challenges to the injured sites [7]. In recent years, there has been a strong emphasis on developing mitochondria-targeting agents. The mitochondrial antioxidant MitoQ is an example of sustained delivery. It has been shown to improve vascular function in healthy adults and is currently being studied in heart failure (NCT03960073) [8].

Recently, mitochondrial-derived vesicles (MDVs) have been identified as a novel mechanism in mitochondrial quality control [9]. In cardiomyocytes, the formation of MDVs is a physiological process that is accelerated by oxidative stress [10]. Moreover, oxidative stress alters the cargo of cardiac MDVs [11,12]. Therefore, MDVs are regarded as a cellular stress response that may play a role in the pathogenesis of CVD. Consequently, MDVs could be addressed therapeutically.

Another therapeutic strategy is the transfer of healthy mitochondria to damaged cells or tissues. Cells depleted of mitochondrial DNA (mtDNA) (ρ^0^ cells) display impaired oxidative phosphorylation since several genes encoding essential subunits of the respiratory chain are part of the mitochondrial genome [13]. Microinjection of isolated mitochondria into ρ^0^ cells improved their respiratory capacity [13]. The first report of mitochondria exchanging between cells in vitro was published in 2006 [14]. In a co-culture approach, mesenchymal stem cells (MSCs) transferred mitochondria to ρ^0^ cells, which exhibited MSC-derived mtDNA. Since then, numerous studies have documented intercellular mitochondrial transfer, both in vitro [15,16,17,18,19] and in vivo [20,21,22,23].

Interestingly, several studies have shown that mitochondrial transfer benefits stressed cardiomyocytes [22,24]. Clinical trials are currently underway to translate this cardioprotective potential into clinical applications. However, there is no comprehensive understanding of the mechanisms underlying mitochondrial transfer. Increasing evidence suggests that mitochondrial cargo is shuttled in extracellular vesicles (EVs), implying that EVs could be used as vectors for mitochondrial transfer [25,26,27]. Furthermore, recent findings are linking MDV and EV biogenesis, suggesting that MDVs are secreted as a subset of EVs [12,28].

This review article will discuss the role of novel mitochondrial mechanisms, focusing on MDVs and mitochondrial transfer via EVs, and will highlight potential therapeutic interventions for CVD.

## 2. The Formation of Mitochondrial-Derived Vesicles Is a Cellular Stress Response

### 2.1. Characterization of Mitochondrial-Derived Vesicles

**Figure 1:**
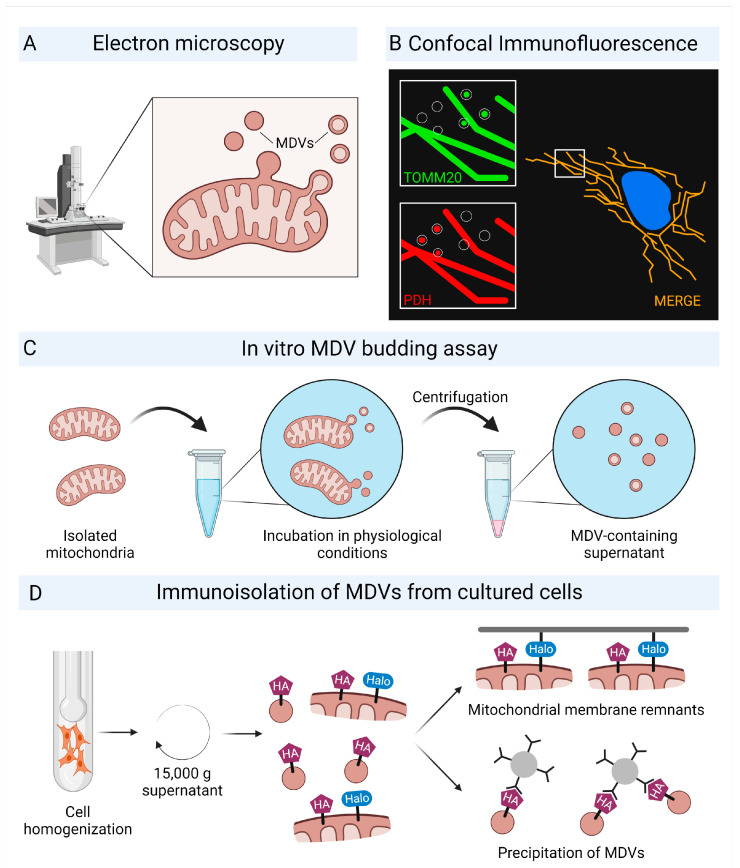
Characterization of mitochondrial-derived vesicles (MDVs). (**A**) Visualization of MDVs by electron microscopy. MDVs with a single membrane form from the outer mitochondrial membrane, whereas MDVs with two membranes form from the inner and outer mitochondrial membranes. (**B**) TOMM20, an outer mitochondrial membrane protein, and PDH, located at the mitochondrial matrix, are two MDV marker proteins for immunofluorescence labeling of MDVs. MDVs are TOMM20 or PDH-positive. White square indicates the merge. (**C**) In vitro MDV budding assay in physiological buffer conditions using isolated mitochondria. (**D**) Immunoisolation of MDVs from cultured cells using hemagglutinin (HA)- and halo-tagging. Created with BioRender.com.

Many unique features of mitochondria are related to their endosymbiotic origin. Mitochondria incorporate mtDNA as a circular genome and contain ribosomes that differ from eukaryotic cells [29]. The capacity to form vesicles is another mechanism that has been retained from their bacterial ancestors [30,31]. Vesicle budding from intracellular mitochondria was first described in 2008 and has since been observed in a variety of cell types and tissues [32]. MDVs are formed in mammalian cells, plants, and yeast, suggesting that MDVs are a conserved mechanism [33,34]. MDVs are single- or double-membrane structures with diameters ranging from 70–100 nm budding from the mitochondrial surface [4,11,35]. Single-membrane MDVs form only from the outer mitochondrial membrane, whereas double-membrane MDVs form from both the inner and outer mitochondrial membranes (Table 1). Electron microscopy can be used to visualize MDVs and their budding (Figure 1A). The MDV cargo is determined by the budding process, which selectively includes mitochondrial proteins. TOMM20, an outer mitochondrial membrane protein required for importing nuclear-encoded proteins, and pyruvate dehydrogenase (PDH) subunits E2/E3bp located at the mitochondrial matrix are two established protein markers commonly used to study MDVs. Two distinct MDV populations can be distinguished based on these proteins: TOMM20-positive, PDH-negative (TOMM20^+^/PDH^−^) and TOMM20-negative, PDH-positive, (TOMM20^−^/PDH^+^) MDVs (Figure 1B) [10,35,36,37,38]. Although PDH-positive vesicles lack TOMM20, they are considered to be composed of inner and outer mitochondrial membrane [9]. MDV cargo selectivity allows them to be distinguished from the mitochondrial network, which is positive for both markers.

The use of marker proteins allows for imaging studies to be performed; however, this implies that MDV characterization is limited to distinct subpopulations. Various subpopulations were investigated, complicating the generalizability of the findings. 

Proteomic approaches enabled a deeper understanding of the MDV cargo. Soubannier et al. developed an in vitro MDV reconstitution assay [11] in which isolated functional mitochondria were incubated under physiologic conditions to generate MDVs (Figure 1C). Following centrifugation, the mitochondria were removed, and the purified MDV-containing supernatant was prepared for proteomic analysis.

A recently developed method allowed for the isolation of MDVs directly from cultured cells. TOMM20^+^ MDVs were isolated from COS7 cells that had been transfected with hemagglutinin-tagged TOMM20 and halo-tagged mitochondrial-anchored protein ligase (MAPL) (Figure 1D). MAPL localizes to the outer mitochondrial membrane but is not found in TOMM20^+^ MDVs. Cells were homogenized, followed by differential centrifugation to remove cell debris and organelles. The remaining supernatant was removed from halo-tagged outer mitochondrial membrane remnants, and TOMM20^+^ MDVs were immuno-isolated using magnetic hemagglutinin-beads [39]. This dual tagging approach enables proteomic analyses of different MDV populations.

### 2.2. Oxidative Stress Increases the Formation of Mitochondrial-Derived Vesicles

Mitochondrial function is crucial for maintaining cellular homeostasis. Therefore, damaged or dysfunctional mitochondria must be efficiently removed. MDVs emerge as an essential quality control mechanism in response to mitochondrial stress, in addition to mitophagy and fission/fusion processes that regulate mitochondrial turnover. Oxidative stress induced by xanthin oxidase [40] or complex III inhibitor antimycin A [41] enhanced the formation of TOMM20^+^/PDH^−^ and TOMM20^−^/PDH^+^ MDVs in kidney fibroblasts [35]. These findings suggest that MDVs are released by mitochondria in response to cellular stress. 

MDV formation has also been observed in the cardiac system. Under physiological conditions, rat cardiomyocytes produced TOMM20^+^/PDH^−^ and TOMM20^−^/PDH^+^ MDVs [10]. Stimulation with antimycin A increased TOMM20^+^/PDH^−^ MDVs without affecting the TOMM20^−^/PDH^+^ MDV count [10]. Doxorubicin, a chemotherapeutic agent known for its cardiotoxic potential [42], increased the generation of TOMM20^+^/PDH^−^ and TOMM20^−^/PDH^+^ MDVs in rat cardiomyocytes [10]. The increase in MDVs preceded adverse effects on cellular respiration, indicating that MDV formation is an early cellular response. Furthermore, doxorubicin-treated C57BL/6 mice exhibited a more than two-fold increase in cardiac MDV budding compared to control mice [10]. 

MDVs may play a role in the cellular response to hypoxia. MDV formation in rat cardiomyocytes increased initially under hypoxic conditions, but decreased with prolonged hypoxia [43]. This time dependence further supports the concept of MDVs as an early response to maintain mitochondrial integrity. 

The specific cargo loading into cardiac MDVs is determined by the type of cellular stress. In vitro generated MDVs of mitochondria isolated from bovine heart tissue treated with antimycin A showed selective incorporation of Core2, a mitochondrial complex III associated protein, while lacking the voltage-dependent anion-selective channel (VDAC), an outer mitochondrial membrane protein. MDVs produced in response to xanthin oxidase stimulation contained VDAC but not Core2 [11]. This suggests that intramitochondrial oxidative stress causes mitochondrial matrix proteins to be loaded into MDVs, whereas extramitochondrial stress causes the incorporation of outer mitochondrial proteins. Furthermore, MDVs were enriched in oxidized proteins compared to the origin mitochondria [11].

Proteomic analysis of MDVs obtained in vitro from isolated rat heart mitochondria provided further insights into the cargo profile of cardiac MDVs [12]. In this study, MDVs generated under baseline or stress conditions were purified using a sucrose gradient and the PDH^+^ MDV-fraction was proteomically analyzed. Among the 250 identified mitochondrial proteins, inner mitochondrial membrane and matrix proteins were found in greater abundance than outer mitochondrial proteins [12]. The cargo of cardiac MDVs was altered by antimycin A-mediated oxidative stress toward matrix proteins associated with the citric acid cycle, amino or fatty acids metabolism, and iron sulfate clusters, indicating a preferential loading of proteins involved in redox processes [12]. Furthermore, antimycin A promoted the loading of proteins containing oxidation-prone hyper-reactive cysteine residues, supporting the hypothesis of selective enrichment of oxidized material in MDVs [12].

In summary, MDV formation in the heart was observed under basal conditions and can be regarded as a physiologic process induced in response to cellular stressors, such as oxidative stress, which further modulates MDV cargo loading. The role of MDVs in human cardiomyocytes or heart tissue as well as the vascular system is still unknown. Further studies are needed to determine the mechanistic role of MDVs in the pathogenesis of CVD.

### 2.3. Biogenesis and Trafficking of Mitochondrial-Derived Vesicles

**Figure 2:**
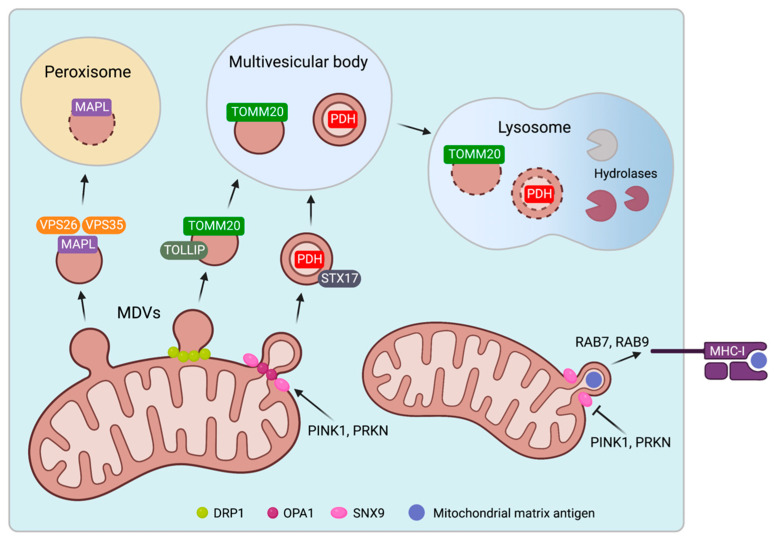
Biogenesis and trafficking of mitochondrial-derived vesicles (MDVs). The formation of specific MDV populations, which are then directed to their cellular destination, is governed by distinct mechanisms. MAPL+ MDVs are recruited to peroxisomes by VPS26 and VPS35. TOMM20^+^/PDH^−^ and TOMM20^−^/PDH^+^ MDVs are shuttled to the endolysosomal system by TOLLIP and STX17. MDVs containing mitochondrial matrix proteins allow for the presentation of mitochondrial antigen on the cell surface. Perforated membranes of the vesicles indicate degradation. Created with BioRender.com.

There are only a few mechanisms suggested to be involved in MDV formation (Figure 2). Several studies have shown that the budding of MDVs is independent of dynamin-related protein 1 (DRP1), a GTPase required for mitochondrial fission, since DRP1 silencing using siRNA had no effect on MDV generation [32,35,36]. However, CRISPR/Cas9-mediated knockout of DRP1 and blocking the recruitment of DRP1 via triple knockout of its receptors prevented the formation of TOMM20^+^ MDVs [39]. The effect of DRP1 deficiency on TOMM20^−^/PDH^+^ MDVs was not investigated in this study.

PTEN-induced kinase 1 (PINK1) and E3 ubiquitin-protein ligase parkin (PRKN), two essential proteins regulating mitophagy [44], were implicated in stress-induced MDV formation. Antimycin A treatment increased the formation of TOMM20^−^/PDH^+^ MDVs in osteosarcoma cells overexpressing PRKN, which was prevented by PINK1 loss-of-function [36]. An impact on the generation of TOMM20^+^/PDH^−^ MDVs was not investigated. The proteome of MDVs derived from PINK1- and PRKN-deficient mouse brains showed no differences compared to MDVs obtained from wild-type mice [45].

Two proteins have been identified to regulate the loading of mitochondrial inner membrane and matrix proteins into MDVs [28]. SNX9, a sorting nexin required for endosomal trafficking [46], was recruited to the mitochondrial surface and drove the formation of MDVs containing matrix proteins. OPA1, a dynamin-like GTPase that localizes in the inner mitochondria membrane and mediates mitochondrial fusion [47], is another protein required for matrix-positive MDVs. Silencing SNX9 or OPA1 reduced the number of matrix-positive MDVs without affecting the formation of TOMM20^+^ MDVs [28].

While mechanisms initiating the MDV formation require further investigation, their intracellular trafficking routes have been characterized in greater detail. MAPL^+^ TOMM20^−^ MDVs were targeted to peroxisomes in HeLa and osteosarcoma cells [32]. The recruitment of the vacuolar protein sorting-associated proteins VPS35 and VPS26, which formed a complex with MAPL, was required for the trafficking of MAPL^+^ MDVs to peroxisomes. VPS35 or VPS26 silencing reduced the co-localization of MAPL^+^ MDVs with peroxisomes [48]. VPS 29, which acts together with VPS35/26 to regulate endosome trafficking toward the Golgi apparatus [49], was not recruited to MAPL^+^ MDVs [48]. 

TOMM20^+^/PDH^−^ and TOMM20^−^/PDH^+^ MDVs can be directed to the endolysosomal system. Inhibition of lysosomal acidification by bafilomycin, a vacuolar H^+^ATPase inhibitor, prevented lysosomal degradation and promoted intracellular accumulation of TOMM20^+^/PDH^−^ and TOMM20^−^/PDH^+^ MDVs [35]. Furthermore, targeting TOMM20^-^/PDH^+^ MDVs to lysosomes required syntaxin 17 (STX17), a SNARE protein that drives membrane fusion processes in complex with other SNARE family members [37]. STX17 was found in MDVs generated in vitro and co-localized with TOMM20^−^/PDH^+^ MDVs. STX17 silencing reduced the number of TOMM20^−^/PDH^+^ MDVs directed to lysosomes. STX17 recruitment to MDVs occurred prior to budding, which was mediated by PINK1 and PRKN [37]. SNARE proteins SNAP29 and VAMP7 are also required for MDV fusion with lysosomes and may form a complex with STX17. Of note, STX17 knockdown had no effect on the trafficking of TOMM20^+^/PDH^−^ MDVs [37]. A recent study showed that TOLLIP, which interacts with endosomal sorting complexes, was involved in the turnover of TOMM20^+^/PDH^−^ MDVs [38]. TOLLIP localized to TOMM20^+^/PDH^-^ MDVs, and its knockdown caused an accumulation of these vesicles, suggesting that their transition into the endolysosomal system was halted. Furthermore, TOLLIP co-localized with PRKN and silencing PRKN reduced TOMM20^+^/PDH^−^ MDV trafficking to lysosomes [38]. 

These studies demonstrate the existence of distinct pathways for directing specific MDV populations to their intracellular destination. Overall, trafficking routes to lysosomes and peroxisomes suggest that MDVs are primarily degraded. Selective cargo loading allows for the removal of damaged proteins, preserving mitochondrial integrity and avoiding the need to remove entire mitochondria.

Almost every cell type expresses intracellular peptides on the cell surface via MHC-I [50], which is a critical physiologic mechanism for preventing autoimmunity against endogenous structures while recognizing damaged cells or intracellular pathogens. MDVs are involved in mitochondrial antigen presentation [51]. RAW cells expressing a fluorescent reporter antigen that localizes to the mitochondrial matrix were used in this study. A specific T-cell-hybridoma, whose activation level indicates the reporter’s abundance on the cell surface, was used to measure the loading of the mitochondrial antigen on MHC-I. Heat stress or lipopolysaccharide (LPS) exposure caused RAW cells to form MDV-like structures that were TOMM2-negative but positive for the matrix-targeted reporter antigen. Concurrently, its presentation on MHC-I increased independently of Drp1, which could be reversed by silencing SNX9 [51]. These findings show that SNX9-dependent MDVs are required to load mitochondrial matrix peptides on MHC-I, while PINK1 and PRKN negatively regulated MDV-mediated mitochondrial antigen presentation. Furthermore, silencing of the small GTPases RAB7 or RAB9 attenuated mitochondrial antigen presentation, implying that they are involved in MDV trafficking [51]. Notably, mechanisms underlying the presentation of mitochondrial outer membrane antigens remain unknown.

## 3. Cellular Release of Extracellular Vesicles with Mitochondrial Cargo

### 3.1. Mitochondrial Cargo Is Present in Extracellular Vesicles

EVs are membrane-bound particles secreted from cells that carry biomolecules, such as proteins, nucleic acids, and lipids [52,53] (Table 2). Exosomes are small EVs that range in size from 30–150 nm. They originate from multivesicular bodies (MVB), an endosomal compartment defined by the presence of intraluminal vesicles. These vesicles form by budding inward into the MVB and are secreted when the MVB membrane fuses with the cell surface [54]. EVs formed by outward budding of the plasma membrane can be larger (up to 1 µm) and are commonly referred to as microvesicles or ectosomes [55]. Moreover, dying cells release apoptotic bodies, which are vesicle-enclosed cell fragments [56,57]. 

Several studies have found mitochondrial content in EVs, establishing a link between EVs and MDVs. Mitochondrial proteins were found in both microvesicles and exosomes released by dendritic cells, with microvesicles having a higher abundance [58]. Flow cytometric analyses of plasma EVs isolated from healthy individuals revealed that all small, mid-sized, and larger EV subsets were partly labeled with MitoTracker, a carbocyanide-based dye that accumulates in active mitochondria [59]. This does not imply that all MitoTracker-positive EVs contain entire mitochondria, which are 0.5–5 µm in size and thus are larger than exosomes [60,61]. Rather, the presence of active mitochondrial enzymes may define small MitoTracker-positive EVs.

Nevertheless, microvesicles as larger EVs were shown to engulf whole mitochondria in LPS-treated monocytes [25] and MSCs [26]. Moreover, microvesicles released by activated platelets displayed oxygen consumption, indicating the presence of functional mitochondria or mitochondrial fragments within vesicles [62]. EVs from human melanoma tissue were found to be enriched in mitochondrial proteins, particularly those derived from the inner mitochondrial membrane. Interestingly, an EV subpopulation positive for MT-CO2 (a component of respiratory chain complex 4) exhibited ATP synthase activity [63]. 

D’Acunzo et al. recently discovered a population of mitochondrial protein-enriched brain-derived EVs [27,64]. A density gradient was used to separate the 100,000 g EV-containing pellet into eight fractions. The highest density fraction was primarily made up of double membrane, electron-dense vesicles, resembled the structure of bilayered MDVs, and selectively contained a variety of inner and outer mitochondrial membrane proteins. This fraction exhibited ATP production capacity—another support for active mitochondrial enzymes in EVs. Exosomal marker proteins, such as Alix or CD63 were absent, indicating that EVs containing mitochondrial proteins are a distinct EV population [27,64].

Beyond mitochondrial proteins, mitochondrial DNA has been identified as an EV cargo. The entire mitochondrial genome was found in plasma-derived EVs from patients with metastatic breast cancer. Cancer-associated fibroblasts could transfer mtDNA via EVs, restoring oxidative phosphorylation in breast cancer cells treated with hormonal therapy [65]. Blood-circulating EVs containing mtDNA have also been reported in healthy individuals, where mtDNA levels within EVs correlated inversely with age [66], which is consistent with an age-related decline in MitoTracker-labeled EVs [59].

### 3.2. Mechanisms of Mitochondrial Cargo Loading into Extracellular Vesicles

Mitochondrial content in exosomes and microvesicles suggests distinct mechanisms of cargo loading and EV release.

**Figure 3:**
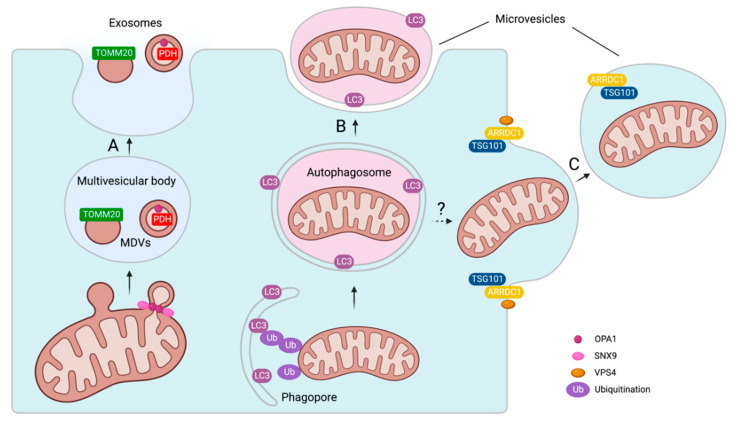
Mechanisms of mitochondrial cargo loading into extracellular vesicles and mitochondrial-derived vesicles (MDVs). (**A**) Fusion of MDV-containing multivesicular body with the plasma membrane leads to an exosomal release of stored MDVs. (**B**) Mitochondria in LC3-positive autophagosomes could be secreted as microvesicles via fusion of the outer phagosomal membrane with the plasma membrane (question mark indicates a possible pathway) or (**C**) through outward budding of the plasma membrane mediated by ARRDC1 and TSG101. Created with BioRender.com.

The mechanisms that drive the inclusion of mitochondrial proteins into exosomes are poorly understood. A hypothesis is that exosomes enriched in mitochondrial proteins share common biogenesis with MDVs. As previously mentioned, bacteria, as mitochondrial progenitors, release vesicles into the extracellular space [31]. Externalization of vesicles facilitates cargo transfer between bacteria. Therefore, MDVs are most likely secreted extracellularly, promoting intercellular communication. Although direct evidence of MDV secretion is still pending, MDVs can localize to MVBs [35]. MVBs can fuse with lysosomes allowing for cargo degradation [67], but they may also serve as a mechanistic basis for MDV externalization via plasma membrane fusion and subsequent exosomal release of stored MDVs into the extracellular space (Figure 3A).

MSCs release mitochondria in microvesicles that are positive for microtubule-associated protein 1 light chain 3 (LC3), an autophagosome marker (Figure 3B,C) [26]. The precise mechanism by which autophagosome-engulfed mitochondria are secreted extracellularly is unknown. Autophagosomes are double-membraned structures, and the fusion of their outer membrane with the plasma membrane would result in the release of single-membrane vesicles (Figure 3B). On the other hand, there is evidence that mitochondria-containing microvesicles form via plasma membrane outward budding (Figure 3C) [26]. This involved arrestin domain-containing protein 1 (ARRDC1), which binds to the plasma membrane via its arrestin domain and recruits tumor suppressor gene 101 (TSG101) to initiate the budding process [26]. The ATPase VPS4 is most likely required for the final scission from the plamsa membrane [68]. ARRDC1 and TSG101 are both found in mitochondria-containing microvesicles, whereas VPS4 has not been identified as a cargo [26]. Mitochondria loading into platelet-derived microvesicles required the actin cytoskeleton and was not dependent on microtubules [62]. Platelet activation causes the formation of long membrane protrusions [69], which may facilitate mitochondrial vesicular secretion.

The overlap of the MDV and EV proteome supports the hypothesis that MDVs are released as a subset of EVs. One study compared the proteomic profile of in vitro-produced MDVs to publicly available EV proteome datasets from the ExoCarta database [12,70]. MDVs reconstituted from rat hearts contained 7.2% of all mitochondrial proteins listed in ExoCarta. Of note, the ExoCarta database contains proteomic data from several species. When the MDV proteome was compared to individual EV datasets, it was discovered that the EV proteome derived from stressed cells overlapped the most with MDV proteins [12]. For example, more than 50% of EV proteins secreted from hypoxia-treated cardiac fibroblasts overlapped with the MDV dataset, and serum-deprived smooth muscle cell-derived EVs had a 40% overlap [12].

There is evidence linking MDV and EV biogenesis on a mechanistic level. SNX9 and OPA1 are involved in the loading of mitochondrial cargo into EVs [28]. Silencing SNX9 or OPA1 with siRNA reduced the number of MDVs enriched for matrix proteins and concomitantly reduced the presence of these proteins in EVs [28]. These findings support the hypothesis that MDVs are an EV subpopulation, demonstrating SNX9 and OPA1 as common mechanisms for producing matrix-positive MDVs and EVs containing mitochondrial matrix proteins. Interestingly, OPA1 was identified as EV cargo, whereas SNX9 was not found within EVs [28]. Of note, TOMM20^+^ MDVs form independently of SNX9 and OPA1 [28]. These findings suggest that SNX9 and OPA1 are primarily responsible for generating MDVs and EVs enriched for matrix proteins, whereas loading outer membrane proteins is dependent on separate processes.

The loading of mitochondrial proteins into exosomes is altered by cellular stress. Stimulating mouse embryonic fibroblasts with antimycin A reduced the inclusion of mitochondrial matrix proteins into exosomes, while increasing the amount of matrix-positive MDVs that co-localized with lysosomes [28]. This suggests that oxidative stress increases the lysosomal routing of MDVs, preventing exosomal release. Interestingly, antimycin A had no effect on the abundance of TOMM20 in exosomes and the co-localization of TOMM20^+^ MDVs with lysosomes [28]. This observation could be explained by the fact that antimycin A causes internal reactive oxygen species (ROS) production while having no effect on outer membrane proteins, such as TOMM20.

### 3.3. Biological Effects of Extracellular Vesicles with Mitochondrial Cargo

EVs transfer mitochondrial content between cells, exerting protective effects on the recipient cells, especially during cellular stress [71,72,73,74,75]. 

Since obesity is a risk factor for CVD, EV-mediated crosstalk between adipose tissue and myocardium may be pathophysiologically relevant. EVs with oxidized mitochondrial content released by palmitate-stressed adipocytes stimulated myocardial ROS production in vitro and in vivo [71]. Interestingly, the vesicular transmission of dysfunctional mitochondria from adipocytes to cardiomyocytes may function as a protective preconditioning signal. In mice, injection of EVs derived from stressed adipocytes prior to coronary artery ligation reduced myocardial injury [71]. This cardioprotective effect was not seen in EVs derived from PRKN^−/−^-adipocytes, which had less mitochondrial EV cargo [71]. 

Further studies investigated cardioprotective effects of mitochondria-loaded EVs. Cardiomyocytes derived from human induced pluripotent stem cells were cultured, and mitochondria-containing EVs were isolated from the conditioned culture medium [74]. When these EVs were applied to hypoxic cardiomyocytes, they restored intracellular ATP levels and improved contractility, whereas isolated naked mitochondria had no effect, suggesting that vesicular packaging facilitated mitochondrial uptake [74]. Another study found that mitochondria-containing EVs derived from MSCs were internalized by human cardiomyocytes in vitro, inhibiting ROS formation and maintaining ATP production after doxorubicin exposure, thereby reducing cardiomyocyte damage [75].

Several studies have provided evidence for an additional function of mitochondria-containing EVs, indicating that the vesicular release of mitochondrial cargo may serve as a mechanism for disposing of damaged mitochondria. Brown adipocytes, stressed by cold exposure, secreted oxidized mitochondrial material in EVs, which was subsequently taken up by resident macrophages [76]. Macrophage depletion resulted in the accumulation of EVs in brown adipose tissue and impaired thermogenesis [76]. 

A similar mechanism has been discovered in the cardiac system. Cardiac resident macrophages phagocytized mitochondria-containing EVs released by cardiomyocytes [23]. The macrophage receptor Tyrosin-protein kinase Mer (MERTK) recognized phosphatidyl-serine on the vesicles’ surface and mediated phagocytic uptake [23]. Stress induced by catecholamine treatment or coronary artery ligation increased the phagocytic clearance of cardiomyocyte-derived EVs with mitochondrial content in mice [23]. Furthermore, mice depleted of cardiac resident macrophages demonstrated diastolic dysfunction and lower survival rates following coronary artery ligation [23]. These findings suggest that tissue-resident macrophages play an important role in maintaining mitochondrial health by removing dysfunctional mitochondria secreted by EVs.

Despite the beneficial effects of EV-mediated mitochondrial transfer, secreted mitochondrial cargo has an immunogenic potential. Extracellular mitochondrial components can act as damage-associated molecular patterns (DAMPs), that are recognized by the innate immune system and trigger inflammatory responses. Mitochondrial DAMPs, such as circular DNA or N-formylated peptides, resemble bacterial motifs and are recognized by pattern recognition receptors [77,78]. Endothelial cells were activated by mitochondria-containing microvesicles derived from LPS-treated monocytes, as evidenced by interleukin-8 secretion and increased expression of cellular adhesion molecules VCAM and ICAM-1 [25]. Interestingly, exosomes containing mitochondrial proteins had no pro-inflammatory effects [28]. A comparison of isolated mitochondria and exosomes from mouse embryonic fibroblasts revealed that mitochondria, but not exosomes, stimulate IL-6 secretion in macrophages [28]. 

The inflammatory effects of secreted mitochondria may impair mitochondrial transfer. However, there is evidence that the inflammatory signaling in mitochondria-ingesting macrophages is inhibited during mitochondrial uptake. Phinney et al. showed that MSC-derived EVs attenuated toll-like-receptor (TLR) signaling in recipient macrophages, which could be mediated by microRNA cargo [26]. This suggests that the immunomodulatory mechanisms of EVs tolerate recipient cells, allowing for mitochondrial uptake.

## 4. Therapeutic Applications of Mitochondria in Cardiovascular Disease

The therapeutic approach of transferring functional mitochondria to damaged cells or tissue, also known as mitochondrial transplantation, is gaining increasing attention. Several studies investigated the regenerative capacity of isolated naked mitochondria in myocardial ischemia. McCully et al. reported for the first time in 2009 that intramyocardial injection of viable mitochondria reduced infarct size in a rabbit model [79]. Large animal studies supported that mitochondrial injection reduces myocardial damage after ischemia-reperfusion [80,81]. In 2017, the first clinical trial with five pediatric patients requiring extracorporeal membrane oxygenation (ECMO) after cardiac ischemia was conducted [82]. During the same surgical procedure, mitochondria were freshly isolated from an autologous skeletal muscle biopsy and injected intramyocardially. The mitochondrial injection caused neither arrhythmia nor cardiac bleeding, indicating that it was a safe method [82]. Myocardial injection of mitochondria necessitates surgical access via thoracotomy, and multiple injections are required to achieve an adequate distribution in the ischemic area [80,81,83], limiting the feasibility of this method.

Therefore, alternative routes of mitochondrial administration were investigated. The use of cardiac catheterization to deliver mitochondria intracoronary is a promising and minimally invasive approach [84,85,86,87]. Since cardiac catheterization and coronary revascularization are standard treatments for acute myocardial infarction, intracoronary administration of mitochondria has a greater potential for clinical use. After successful revascularization, mitochondria could be applied to the affected coronary artery to reduce ischemia-reperfusion damage. Intracoronary application of mitochondria reduced infarct size and improved ventricular function in porcine models [85,86]. Nanoparticle labeling and positron emission tomography demonstrated that the myocardium took up mitochondria after intracoronary administration [84,85].

Aside from myocardial ischemia, clinical trials in various fields of medicine are currently underway, treating patients with isolated mitochondria (Table 3).

## 5. Perspective

There have been no clinical studies using EVs as mitochondrial transfer vectors. The cardioprotective effects of EV-mediated mitochondrial transfer observed in vitro and in mouse models suggest that EVs have therapeutic potential as a mitochondria delivery system. When compared to naked mitochondria, vesicular transmission of mitochondria may offer significant advantages. EVs may aid in mitochondrial uptake. Mitochondria-containing EVs improved bioenergetics in hypoxia-injured cardiomyocytes and left ventricular function in a mouse model of myocardial infarction [74]. This was not observed after injection of isolated naked mitochondria, strengthening the idea that vesicular delivery increases mitochondrial uptake [74].

In perspective, EVs could be modified to transport mitochondrial cargo in a highly targeted manner. The bioengineering of EV surface molecules will allow for specific binding to target cells, allowing for intravenous application [88]. Since mitochondria in EVs were more resistant to oxidative stress and calcium overload than isolated mitochondria, vesicular packaging may improve mitochondrial viability and stability [74]. Furthermore, mitochondria that have been engulfed in EVs may have a lower immunogenic potential. However, there is evidence that the inflammatory effects of mitochondrial content are unrelated to vesicular packaging. In an in vitro study, disrupting vesicular integrity by sonication did not reduce the immunogenic effects of mitochondria-containing EVs in endothelial cells [25]. Nonetheless, the immunomodulatory effects seen in mitochondria-ingesting macrophages could be translationally utilized [26,89]. MicroRNAs or other molecules could be added as EV cargo to improve mitochondrial uptake of the recipient cells.

Since mitochondrial transmission via EVs may provide significant advantages over naked mitochondria, large animal and clinical trials should be conducted in the future to assess the safety and efficacy of EV-mediated mitochondrial transfer.

In addition to the transfer of whole mitochondria, the transport of mitochondrial proteins in MDVs has the potential to be therapeutic. In a recent study, hypoxic rat cardiomyocytes were treated with in vitro reconstituted MDVs. These MDVs were taken up and localized to the mitochondrial network, where they reduced ROS production and protected cardiomyocytes from hypoxia-induced damage [43]. These cardioprotective effects imply that MDVs generated in vitro under physiological conditions could be used as therapeutic EVs to deliver functional mitochondrial proteins, thereby improving mitochondrial function in recipient cells. In perspective, the cargo of these MDVs could be modified to transport specific proteins in a highly targeted manner.

In addition to their therapeutic potential as drug delivery vehicles, blood-circulating EVs have gained increasing interest as diagnostic biomarkers in CVD [90,91,92,93]. The specific cargo signature and stability of EVs make them a valuable diagnostic tool. Since mitochondrial dysfunction is a common feature of CVD, detecting mitochondrial cargo in EVs would be an intriguing diagnostic strategy [2]. MDVs have not yet been studied as biomarkers. MDVs, which are secreted as a subset of EVs, could potentially be detected in the blood to assess mitochondrial health in the cardiovascular system. Since MDV cargo changes in response to oxidative stress, circulating MDVs could indicate mitochondrial dysfunction [11,12].

Despite mounting evidence that MDVs are secreted as a subpopulation of EVs, the underlying mechanisms are still unknown. More research into the link between MDVs and EVs is required to fully exploit the diagnostic and therapeutic potential of MDVs. Numerous clinical studies are currently underway to investigate EVs as diagnostic and therapeutic agents, highlighting the future potential of vesicle-based therapies [94].

## Figures and Tables

**Table 1:** Different types of mitochondrial-derived vesicles (MDVs).

	Single-Membrane MDVs	Double-Membrane MDVs
	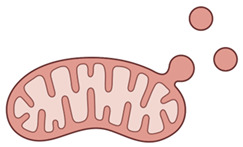	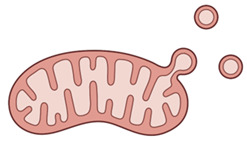
Origin	Budding from the outer mitochondrial membrane	Budding from the inner and outer mitochondrial membrane
Cargo	Outer mitochondrial membrane proteins	Inner mitochondrial membrane and matrix proteins
Protein marker	Translocase of outer mitochondrial membrane 20 (TOMM20), Mitochondrial-anchored protein ligase (MAPL)	Pyruvate dehydrogenase subunits E2/E3bp (PDH), NADH:ubiquinone oxidoreductase subunit A9 (NDUFA9), mitochondrial stress 70 protein (mtHSP70)

images generated with Biorender.com.

**Table 2:** Comparison of mitochondrial-derived vesicles (MDVs) and other classical extracellular vesicles.

Type of Vesicles	Size	Origin/Mechanism of Secretion	Cargo
MDVs	70–100 nm	Budding from mitochondrial surface/ fusion of multivesicular bodies with plasma membrane?	Mitochondrial proteins
Exosomes	30–150 nm	Endolysosomal system/ fusion of multivesicular bodies with plasma membrane	Proteins, lipids, nucleic acids
Microvesicles	100–1000 nm	Outward budding of the plasma membrane	Proteins, lipids, nucleic acids, organelles
Apoptotic bodies	>1000 nm	Cell shrinkage and fragmentation	Organelles, nuclear fragments

**Table 3:** Clinical trials using mitochondrial transfer.

Condition/ Disease	Mitochondrial Administration	Origin of Mitochondria	Status	Trial Identifier
Infertility	Injection into oocytes during in vitro fertilization	Autologous (ovarian stem cells)	Completed	NCT02586298
Extracorporeal membrane oxygenation complication	Intramyocardial injection during surgery/intracoronary infusion with catheter	Autologous (skeletal muscle)	Recruiting	NCT02851758
Cerebral ischemia	Endovascular infusion with catheter	Autologous (skeletal muscle)	Recruiting	NCT04998357
Polymyositis or dermatomyositis	Intravenous infusion	Allogeneic (umbilical cord-derived MSCs)	Recruiting	NCT04976140

ClinicalTrials.gov—last accessed on 20 December 2022. MSCs: Mesenchymal stem cells.

## Data Availability

Not applicable.
